# Specific Cues Can Improve Procedural Learning and Retention in Developmental Coordination Disorder and/or Developmental Dyslexia

**DOI:** 10.3389/fnhum.2021.744562

**Published:** 2021-12-15

**Authors:** M. Blais, M. Jucla, S. Maziero, J. -M. Albaret, Y. Chaix, J. Tallet

**Affiliations:** ^1^Toulouse NeuroImaging Center, Toulouse University, Inserm, UPS, Toulouse, France; ^2^EuroMov Digital Health in Motion, Univ Montpellier, IMT Mines Alés, Montpellier, France; ^3^Laboratory of Neuro Psycho Linguistics, University of Toulouse, Toulouse, France; ^4^Children’s Hospital, CHU Purpan, Toulouse, France

**Keywords:** serial reaction time task (SRTT), comorbidity, DCD, retention, selective attention

## Abstract

The present study investigates procedural learning of motor sequences in children with developmental coordination disorder (DCD) and/or developmental dyslexia (DD), typically-developing children (TD) and healthy adults with a special emphasis on (1) the role of the nature of stimuli and (2) the neuropsychological functions associated to final performance of the sequence. Seventy children and ten adults participated in this study and were separated in five experimental groups: TD, DCD, DD, and DCD + DD children and adults. Procedural learning was assessed with a serial reaction time task (SRTT) that required to tap on a specific key as accurately and quickly as possible when stimuli appeared on the screen. Three types of stimuli were proposed as cues: the classical version of the SRTT with 4 squares aligned horizontally on the screen, giving visuospatial cues (VS cues), and two modified versions, with 4 letters aligned horizontally on the screen (VS + L cues) and letters at the center of the screen (L cues). Reaction times (RT) during the repeated and random blocks allowed assessing three phases of learning: global learning, specific learning and retention of the sequence. Learning was considered as completed when RT evolved significantly in the three phases. Neuropsychological assessment involved, among other functions, memory and attentional functions. Our main result was that learning and retention were not influenced by the available cues in adults whereas learning improved with specific cues in children with or without neurodevelopmental disorders. More precisely, learning was not completed with L cues in children with neurodevelopmental disorders. For children with DD, learning was completed with the VS and VS + L cues whereas for children with DCD (with or without DD), learning was completed with combined VS + L cues. Comorbidity between DD and DCD had no more impact on procedural learning than DCD alone. These results suggest that learning depends on the nature of cues available during practice and that cues allowing learning and retention depend on the type of disorder. Moreover, selective attention was correlated with RT during retention, suggesting that this neuropsychological function is important for procedural learning whatever the available cues.

## Introduction

Procedural learning is essential in many daily-activities for children and adults, such as wearing, eating, playing music and videogames, using a keyboard, playing sports, writing, etc. Procedural learning is acquired incrementally with training, through repeated exposures to stimuli (Squire and Zola, 1996). Procedural perceptual-motor learning (PPML) subserves the learning of new, and the control of established, sensorimotor skills, rules and habits (Ullman, 2004; Knowlton et al., 2017). Previous research on age-related changes in PPML reported mixed findings. Pioneering studies found comparable performance in children and young adults (Meulemans et al., 1998), while some recent evidence suggests age-related differences in learning. Some of these more recent studies showed that young adults outperform children (Thomas et al., 2004; Hodel et al., 2014; Lukács and Kemény, 2015), while others found better learning performance in children than in adults (Janacsek et al., 2012; Nemeth et al., 2013). The theoretical frameworks of age-related changes also reflect this heterogeneity (Juhasz et al., 2019; Zwart et al., 2019 for a review) but the recent study strongly support models of age-related changes in typical development (Zwart et al., 2019). In summary, both empirical findings and theoretical frameworks present a puzzle of age-related differences in procedural learning.

If we consider the model of Doyon et al. (2003), there are two types of procedural learning: learning by perceptual-motor adaptation, which depends on the cortico-cerebellar loop and learning by memorizing perceptual-motor sequences, which depends on the cortico-striatal loop. According to Nicolson and Fawcett’s (2007) hypothesis, procedural learning would be deficient in children with neurodevelopmental disorders such as Developmental Coordination Disorder (DCD) and Developmental Dyslexia (DD) who are supposed to present with a dysfunction of the cortico-striatal or cortico-cerebellar network, respectively. Despite adequate intellectual abilities, normal sensory abilities, conventional instruction, sociocultural opportunity, and school education, DCD is characterized by impaired motor skills and affecting about 5–6% of school-aged children (American Psychiatric Association [APA], 2013; Wilson et al., 2017; Trainor et al., 2018) and DD present with reading deficits and affects about also 5–6% of school-aged children (American Psychiatric Association [APA], 2013). There is firm evidence of overlap between these two disorders, with rates of comorbidities ranging from 30 to 50% (Chaix et al., 2007; Flapper and Schoemaker, 2013). This overlap has led to the hypothesis of a common impairment of the procedural learning system in DCD and DD (Nicolson and Fawcett, 2007).

Few studies have investigated procedural learning in DCD and DD but no study has compared the motor procedural learning between DCD and DD. Firstly, results suggest that DCD would affect sequential PPML which depends on the cortico-striatal system whereas DD would affect visuomotor adaptations and language-based procedural learning system which dependent on the cortico-cerebellar loop (Ramus et al., 2003; Ramus, 2004; Ullman, 2004; Nicolson and Fawcett, 2007). Even if both loops are supposed to interact at the beginning of practice motor tasks, the model of Nicolson and Fawcett suggests that sequential PPML would be primarily impaired in DCD and secondary impaired in DD (Nicolson et al., 2010). Globally, experimental studies examining PPML do not show a clear picture neither in children with DCD (Wilson et al., 2003; Gheysen et al., 2011; Lejeune et al., 2013; Jarus et al., 2015; Blais et al., 2017, 2018, 2021; Lê et al., 2021) either in children with DD (Vicari et al., 2003, 2005; Stoodley et al., 2008; Deroost et al., 2010; Menghini et al., 2010; Jiménez-Fernández et al., 2011; Yang and Bi, 2011; Hedenius et al., 2013; Lum et al., 2013; Yang et al., 2013; Vakil et al., 2015; Staels and Van den Broeck, 2017; West et al., 2018 for review).

Procedural perceptual-motor learning of sequences is traditionally assessed using Serial Reaction Time Task (SRTT) that allows assessing *global learning* with repetition, as well as *specific learning* of the sequence and its *retention* (Nissen and Bullemer, 1987; Knopman and Nissen, 1991). The only 3 experimental studies using SRTT in DCD (Wilson et al., 2003; Gheysen et al., 2011; Lejeune et al., 2013) have reported inconsistent results, only Gheysen ‘s study reporting no deficit. Similarly, the 14 experimental studies using SRTT in DD have reported inconsistent results (Lum et al., 2013). Some studies indicated that children with DD present an intact procedural learning (Deroost et al., 2010; Menghini et al., 2010; Yang et al., 2013; Vakil et al., 2015; Staels and Van den Broeck, 2017) while others do not (Vicari et al., 2003, 2005; Stoodley et al., 2008; Jiménez-Fernández et al., 2011; Yang and Bi, 2011; He and Tong, 2017). The recent meta-analysis of Clark and Lum (2017) on PPML in neurodevelopmental disorders revealed comparable levels of PPML impairment in DCD and DD (Clark and Lum, 2017). As regard to the comorbidity DCD + DD, a previous study by Biotteau et al. (2015) tested the automatization deficit in the DCD and DD by measuring the performance using a dual task paradigm before and after the practice of a motor sequence task. The results suggested that children with DCD didn’t have more automatization difficulties than those with DD or DCD + DD. However, the absence of a control group limits the conclusion of this study (Biotteau et al., 2015).

Some hypotheses could explain contradictions about PPML in DCD and DD. The complexity of the motor response seems to be important to take into account (in particular the bimanual or unimanual nature of the task to be memorized for the DCD) (Tallet et al., 2015; Blais et al., 2017). The nature of the stimuli could also impact learning. For example, our recent studies highlight that the modality of stimuli affects learning of a temporal sequence in DCD (Blais et al., 2021; Lê et al., 2021). This is in line with the suggestion of previous studies of a multiple system of procedural learning since they point to learning differences with a preservation or a degradation of learning depending on the modality of the stimuli (e.g., sound, verbal, visual) (Goschke et al., 2001; Conway and Christiansen, 2006; Gabriel et al., 2015). For example, Goschke et al. (2001) showed that Broca’s aphasics were selectively impaired in learning the auditory phoneme sequence compared to spatio-motor sequence. Moreover, Gabay et al. (2012) studied the procedural learning in adults with DD using a SRTT with different stimuli modalities: one corresponded to a classic visuospatial sequence and the other corresponded to a sequence of letters. Their results revealed that, while control adults learned both the visuospatial sequence and the letter sequence, adults with DD presented a deficit for learning of the sequence of letters but a preservation for learning of the visuospatial sequence. The practical conditions that could influence learning have not been developed in studies.

Part of the discrepancy in these results could be explained by neuropsychological variables involved in procedural learning tasks. Lum et al. (2019) suggest that learning a visuospatial sequence can modulate levels of visual attention of participants. Specifically, it seems that, as sequence is learned, fewer demands are placed on visual attentional resources (Lum et al., 2019). This result deserves to be examined in the light of difficulties in executive functions of both DCD and DD children. Several studies show a specific deficit in working memory in DCD, especially for the visuospatial component of the working memory (Alloway and Temple, 2007; Alloway, 2011; Tsai et al., 2012). In DD, studies including visuospatial working memory assessment show inconsistent results: with impaired (Menghini et al., 2010) or preserved (Gould and Glencross, 1990; Kibby et al., 2004) abilities. In a large number of studies, there is a correlation between visuospatial working memory and the level of motor impairment assessed by MABC-2 suggesting a link between the low visuospatial working memory and the low motor level (Michel et al., 2011; Rigoli et al., 2012; Pratt et al., 2014). van Cappellen–van Maldegem et al. (2018) demonstrated that in a throwing motor learning task, children with DCD with better visuospatial working memory capacity improved their throwing accuracy more than children with lower visuospatial working memory capacity. The visuospatial working memory could therefore be a serious candidate explaining a deficit in procedural learning of a sequence. According to attention, Staels and Van den Broeck (2017) showed that DD children’s speed during implicit and explicit serial reaction tasks did not significantly differ from that of control children when controlled for attentional level (measured by ADHD questionnaire).

On this basis, the first objective of the present study is to test a possible deficit in PPML using a SRTT in DCD and/or DD, compared to typically-developing children. Given that the development of PPML is still in debate, we also include healthy adults in the study. Based on the idea that the nature of the stimuli has an impact on PPML in DCD and in DD, we also aim to test the effect of the stimuli on PPML of the sequence by presenting a visuospatial sequence and/or a sequence of letters. Our general hypothesis is that adults as well as typically-developing children will learn in all conditions. Moreover, we hypothesize that DCD children (with or without DD) will present a deficit in PPML in visuospatial sequence. We also hypothesize that PPML of a sequence of letters would be impaired in children with DD (with or without DCD). Finally, we hypothesize that PPML would be impaired for the sequence of combined visuospatial and letter stimuli only for the comorbid group of DCD and DD. The second objective of this study is to explore whether neuropsychological variables are linked to the final performance of learning of the motor sequence regardless the group. Our assumptions are as follows: verbal working memory (assessed by digit span) could be particularly implied in procedural learning with verbal stimuli (Letters), visuo-spatial functions and visuospatial working memory (assessed by judgment of line and corsi block-tapping task) could be particularly implied in procedural learning with visuospatial stimuli (VS). More exploratory, attention (assessed by the *d2* test) and tactile recognition of fingers could be linked with the procedural learning in all conditions given that we proposed a bimanual task.

## Materials and Methods

### Participants

Seventy children between 8 and 12 years old (10.08 ± 1.19 years): 20 TD children, 11 children with DCD, 24 children with DD, and 15 children with DCD + DD and 10 adults (7 women) aged between 25 and 35 years participated in the study (see Table 1 for characteristics). They were all right-handed (mean = 93 ± 10), as assessed by the Edinburgh Handedness Inventory (Oldfield, 1971). Participants had corrected-to-normal vision and hearing. We did not include participants who had a regular musical practice, as verified by pre-experimental questionnaire as it could have an impact on the performance. The TD and DD children and their parents reported no perceptual-motor disorder, no psychomotor therapy, and their total impairment Movement Assessment Battery for Children score (M-ABC, Soppelsa and Albaret, 2004) had to be above the 15th percentile (mean score for TD = 3.92 ± 3.08; and mean score for DD = 4.95 ± 2.77). DCD and DCD + DD children had a total M-ABC score below the 5th percentile (mean score for DCD = 21.41 ± 5.02; and mean score for DCD + DD = 20.78 ± 5.90). None of children have intellectual disability verified by 2 subtests of the WISC-IV (Wechsler, 2005) and no children have comorbidities with other neurodevelopmental disorder (attention deficit/hyperactivity disorder or autism spectrum disorder). Adults reported no perceptual-motor disorder. 7 DCD, 14 DD, 4 DCD + DD, and 3 TD children were not included due to non-matching with inclusion criteria. Three children (1 TD, 1 DCD, and 1 DD) were excluded from the protocol because the instructions were not respected.

**TABLE 1:** Means and standard deviation of scores of neuropsychological tests of all groups.

	Adults (*n* = 10; 7♀)	TD (*n* = 20; 10♀)	DCD (*n* = 11; 4♀)	DD (*n* = 24; 12♀)	DCD + DD (*n* = 15; 4♀)
Age	26.9 (3.81)	10.17 (1.30)	9.78 (1.13)	10.06 (1.07)	10.36 (1.24)
Manual laterality	96 (8.43)	93.50 (9.88)	93.64 (11.20)	92.50 (10.12)	92.00 (10.14)
M-ABC	—	3.92 (3.08)	21.41 (5.02)***	4.95 (2.77)	20.78 (5.90)***
Similarities WISC-IV	—	12.7 (2.93)	12.45 (3.93)	11.83 (3.84)	12.07 (2.55)
Picture Concept WISC-IV	—	10.15 (2.05)	9.63 (1.85)	10.21 (1.96)	9.20 (1.37)
Verbal forward working memory	—	11 (3.17)	9 (1.92)***	8 (1.79)***	9 (2.79)***
Visuospatial forward working memory	—	56 (9.30)	40.73 (8.71)***	53.33 (9.17)	44.33 (8.22)***
Verbal backward working memory	—	11.4 (3.20)	9.27 (2.57)	6.87 (2.17)***	7 (1.96)***
Visuospatial backward working memory	—	55.80 (6.69)	47.36 (8.38)***	52.50 (6.40)	49.13 (7.54)***
Tactile Recognition of Digits	—	16.4 (0.99)	14.25 (2.13)***	15.5 (1.78)	16.03 (1.06)
Visuospatial abilities	—	0.50 (0.63)	−0.84 (0.91) ***	−0.33 (1.09)***	−0.54 (0.82)***
Attention score	—	297.50 (51.56)	223 (74.08) ***	256.68 (67.72)***	252.93 (52.17)***

TD, typically-developing children; DCD, developmental coordination disorder; DD, developmental dyslexia; M-ABC, movement assessment battery for children; WISC-IV, Wechsler intelligence scale for children. ***p < 0.016: comparison of the results of each group with neurodevelopmental disorder (DCD, DD, DCD + DD) with the results of the TD group.

The study was in agreement with the ethical standards laid down in the declaration of Helsinki. The protocol was promoted by the national Ethical Committee of the Institute for Medical Research (Inserm, 2014-A01239-38). Characteristics of participants are detailed in Table 1.

### Materials

In the experiment, a computer with Presentation software (Version 18.0, Neurobehavioral Systems, Inc., Berkeley, CA^1^) was in front of the experimenter. This computer delivered visual instructions and visual stimuli to a connected 24″ screen located 80 cm in front of the participants. The participant’s responses were collected using the same software by a computer keyboard placed in front of the participant. The response keys on the keyboard were “D,” “F,” “G,” or “H” and were provided with a colored sticker. It was not specified to the participant which letter were placed under the stickers. All other keys on the keyboard were hidden.

### Tasks

#### Neuropsychological Tasks

• **Tactile Recognition of Fingers:** Participant have to put one hand down on the table. The participant’s hand was hidden. Experimenter touch one or two fingers simultaneously on the second phalanx and participant have to localize digital tactile stimuli. There were 18 stimuli for right hand and 18 stimuli for left hand. The aim of this task was we make sure that children distinguished their fingers. The number of digits correctly localized was collected.

• WISC IV **Digit span (Wechsler, 2005)** was used as a measure of Working memory. Children must repeat numbers in the same order as presented aloud by the examiner (forward Digit span) or in the reverse order of the one presented by the examiner (backward Digit span). The number of digits correctly remembered was collected and standardized for age (standard note).

• WISC IV **Corsi Block-Tapping Task (Wechsler and Naglieri, 2009)** measures visuospatial and working memory. Participants were asked to observe the sequence of blocks “tapped” and then repeated the sequence in the same (forward) or the reverse (backward) order. The number of blocks correctly remembered was collected and standardized for age (T note).

• **Attention *d2* test (Brickenkamp, 1998):** The *d2* test measure of selective and sustained attention and visual scanning speed. This paper and pencil test asks participants to cross out any letter “d” with two marks around above it or below it in any order. The surrounding distractors are usually similar to the target stimulus, for example a “p” with two marks or a “d” with one or three marks. The number of responses and the errors were collected. The final variable GZ-F is the subtraction between the number of responses processed and the errors.

• **Judgment of Line Orientation (JLO) (Benton et al., 1983)** measures a person’s visuospatial ability to match the angle and orientation of lines in space. Subjects are asked to match two angled lines to a set of 11 lines that are arranged in a semicircle and separated 18 degrees from each other. The number of correct responses was collected and standardized for age and gender (standard deviation note).

#### Experimental Task

**FIGURE 1:**
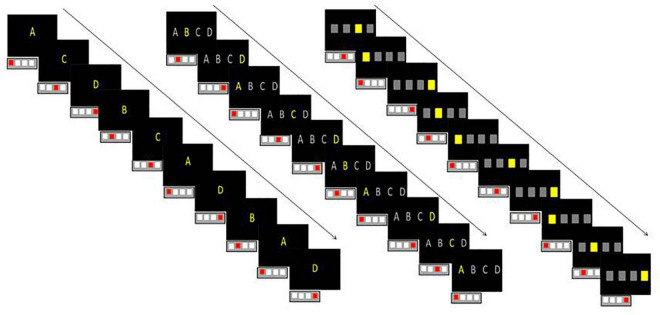
Schematic representation of the three conditions (Letter on the left, Visuospatial + Letter in the middle, and Visuospatial on the right) when displaying the three sequences with the corresponding motor response schematized by a keyboard with four keys.

The participant was instructed to answer as quickly and accurately as possible by pressing the “D”, “F”, “G,” or “H” key on the computer keyboard when one of the 4 visual stimuli appeared in 3 conditions:

• *Letter* condition in which one of the four letter A, B, C, or D was presented in the center of the screen (Figure 1 left). Participant had to press D, F, G, or H key when the letter A, B, C, or D, respectively, appeared.

• *Visuospatial* + *Letter* condition in which the four letters (spaced from 3 cm) A, B, C, et D were displayed linearly and one of them appeared in yellow (Figure 1 middle). Participant had to press D, F, G, or H key when the letter A, B, C, or D, respectively, appeared in yellow.

• *Visuospatial* condition in which four squares (spaced from 3 cm) were displayed linearly and one of them appeared in yellow (Figure 1 right). Participant had to press the D, F, G or H key when the 1st, 2nd, 3rd, or 4th square, respectively, appeared in yellow.

Responses were performed with the index and middle finger of both hands on the 4 keys D, F, G, and H on the computer keyboard hidden under post it. As soon as a response was given or after a time of 3000 ms without response, the following stimulus appeared after a time interval of 250 ms.

In each of the 3 conditions, a determined sequence of stimuli was repeated without the participant being informed. Three different sequences of 10 stimuli were therefore necessary for this task (one sequence per condition). We have taken the sequence of Wilson et al. (2003) and Gheysen et al. (2011) (1-3-4-2-3-1-4-2-1-4), where 1, 2, 3, and 4 correspond to the location of the square or the appearance of the letters “A,” “B,” “C,” and “D,” respectively. We have also taken the sequence of Lejeune et al. (2013) (2-4-1-3-4-2-1-4-3-1) and we have designed a third sequence (3-1-4-2-1-3-4-1-2-4) according to the same rules present in the first two sequences: the location 1 and 4 of the squares (or the letters “A” and “D”) were displayed 3 times and location 2 and 3 squares (or the letters “B” and “C”) were displayed twice. There was never a succession of the same stimulus nor consecutive triplet (1-2-3 or 4-3-2 for example) too easily detectable.

### Procedure

Participants were invited to sit in a chair in a quiet room with their head 80 cm from the screen. First, participants performed neuropsychological test. Then, participants performed the 3 conditions of experimental task with 6 blocks in each condition (5th block was randomized).

For each condition, a sequence of 10 stimuli was repeated 10 times continuously to form a block (1 block = 100 stimuli). Six block were performed. The first four blocks included the repeated sequence (B1–B4). The fifth block (B5) did not have a repeat sequence and the location of the stimuli was pseudo-randomized in order to the four locations appeared with the same frequency as in the learning sequence (repeated sequences): 1 and 4 (or “A” and “D”) were displayed 30% of the time; 2 and 3 (or “B” and “C”) were displayed 20% of the time. Finally, the sixth block (B6) was similar to the first four with the repeated sequence.

The order of the conditions was counterbalanced as well as the sequences attributed to each condition.

### Data Analysis

Two variables were computed:

The reaction time (RT) corresponded to the time interval between the presentation of the stimulus and the recording of the response. RT was expressed in milliseconds. Only the correct responses were taken into account for the calculation of the RT.

The number of errors corresponded to the number of responses produced that did not match to required response.

### Statistics

To test our primary objective, non-parametric analyses on RT (and possibly errors) were carried out between different block for each group (adults, TD, DCD, DD, and DCD + DD) and each condition (Letter; Visuospatial + Letter; Visuospatial) according to the learning processes:

• *Global learning* was tested with Friedman ANOVA between Block 1 and Block 6 and was indicated by the global change of RT (and possibly errors).
• *Specific learning* was tested with Wilcoxon test between Block 4 and Block 5 and was indicated by the increase of RT (and possibly errors).
• *Retention* was tested with Wilcoxon test between Block 5 and Block 6 and was indicated by the decrease of RT (and possibly errors).

To test our secondary objective, multiple regression analyses were conducted with condition of learning of the block 6 as the outcome and seven neuropsychological scores as predictors and one binary predictor variable representing children with disorder vs. non-disordered to control for the influence of diagnostic status. One multiple regression was conducted for each dependent variable on all groups to increase the sample size.

Three collinearity diagnostics tested with the variance inflation factors indicated that there were no problems with multicollinearity (VIF < 2).

## Results

**FIGURE 2:**
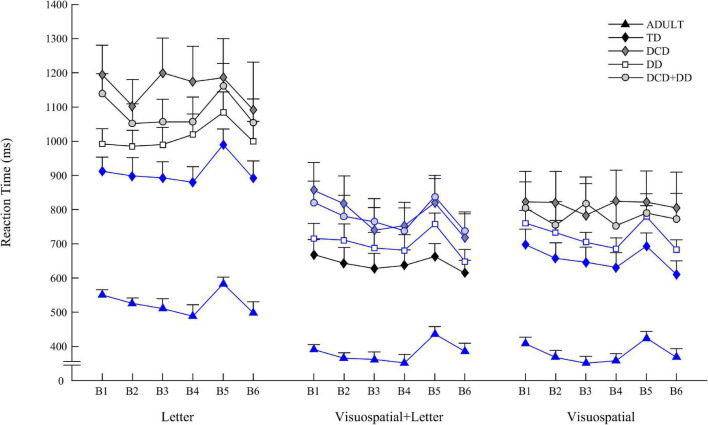
Mean reaction time of adults (black triangle), TD group (black diamond), DCD (gray diamond), DD (white square), and DCD+DD (gray circle) groups for Blocks 1–6 on Letter condition (left) Visuospatial + Letter condition (middle), and Visuospatial condition (right). Blue lines correspond to intact procedural learning. Vertical bars represent inter-individual variability (standard errors).

All the results on global learning, specific learning, and retention, are summarized in the Table 2 and Figure 2. In the text, results of the specific learning and retention were reported only when the global learning is significant. We report the Kendall’s coefficient of concordance as effect size measures.

**TABLE 2:** Results on global learning, specific learning, and retention for each group and condition.

Groups	Learning phase	Letter *p*	Visuospatial+letter *p*	Visuospatial *p*
ADULTS	*Global learning*	**0.001**	**0.000**	**0.002**
	*Specific learning*	**0.006**	**0.005**	**0.006**
	Retention	**0.005**	**0.006**	**0.006**
TD	*Global learning*	**0.008**	0.004	**0.000**
	*Specific learning*	**0.001**	ns	**0.01**
	*Retention*	**0.002**	0.000	**0.002**
DCD	*Global learning*	ns	**0.001**	ns
	*Specific learning*	ns	**0.050**	ns
	*Retention*	ns	**0.012**	ns
DD	*Global learning*	0.03	**0.004**	**0.000**
	*Specific learning*	ns	**0.004**	**<0.001**
	*Retention*	0.011	**<0.001**	**<0.001**
DCD + DD	*Global learning*	ns	**0.001**	0.039
	*Specific learning*	0.046	**0.002**	ns
	*Retention*	ns	**0.004**	ns

*Blue values correspond to intact procedural learning. Black values correspond to procedural learning deficit (when 3 non-significant results appeared in the same condition) or procedural learning difficulties (when 1 or two non-significant results appeared in the same condition).*

### Letter Condition

In the Letter condition, Friedman ANOVA revealed a difference in RT between Block 1 and Block 6 for adults [χ^2^(5) = 19.448, *p* = 0.001, *W* = 0.39], for TD [χ^2^(5) = 15.4, *p* = 0.008, *W* = 0.15], and for DD [χ^2^(5) = 12.31, *p* = 0.03, *W* = 0.10]. Wilcoxon post-tests revealed an increase of RT between B4 and B5 (specific learning) for adults [*Z*(10) = 2.70, *p* = 0.006], for TD [*Z*(20) = 3.21, *p* = 0.001], and also a decrease of RT between B5 and B6 (retention) for adults [*Z*(10) = 2.80, *p* = 0.005], for TD [*Z*(20) = 2.98, *p* = 0.002], and DD [*Z*(24) = 2.51, *p* = 0.01].

Regarding the number of errors, in the letter condition, Friedman ANOVA revealed a difference in the number of errors between Block 1 and Block 6 for adults only [χ^2^(5) = 14.622, *p* = 0.012, *W* = 0.29]. Wilcoxon post-tests revealed an increase of RT between B4 and B5 (specific learning) for adults [*Z*(10) = 2.59, *p* = 0.009] and a decrease of RT between B5 and B6 (retention) for adults [*Z*(10) = 2.65, *p* = 0.008].

### Visuospatial and Letter Condition

In the Visuospatial and Letter condition, Friedman ANOVA revealed a difference in RT between Block 1 and Block 6 for adults [χ^2^(5) = 29.37, *p* < 0.001, *W* = 0.59], for TD [χ^2^(5) = 17, *p* = 0.004, *W* = 0.17] for DCD [χ^2^(5) = 19.78, *p* = 0.001, *W* = 0.36], for DD [χ^2^(5) = 17.21, *p* = 0.004, *W* = 0.14], and for DCD + DD [χ^2^(5) = 20.52, *p* = 0.001, *W* = 0.27].

Wilcoxon post-tests revealed an increase of RT between B4 and B5 (specific learning) for adults [*Z*(10) = 2.80, *p* = 0.005], for DCD [*Z*(20) = 1.95, *p* = 0.050], for DD [*Z*(24) = 2.85, *p* = 0.004], and for DCD + DD [*Z*(15) = 3.01, *p* = 0.002].

Wilcoxon post-tests revealed a decrease of RT between B5 and B6 (specific learning) for adults [*Z*(10) = 2.70, *p* = 0.006], for TD [*Z*(20) = 3.32, *p* < 0.001], for DCD [*Z*(20) = 2.48, *p* = 0.012], for DD [*Z*(24) = 4.25, *p* < 0.001], and for DCD + DD [*Z*(15) = 2.83, *p* = 0.004].

Regarding the number of errors, in the visuospatial and letter condition, Friedman ANOVA revealed a difference in the number of errors between Block 1 and Block 6 for DD only [χ^2^(5) = 21.56, *p* < 0.001, *W* = 0.17]. Wilcoxon post-tests revealed an increase of RT between B4 and B5 (specific learning) for DD [*Z*(24) = 2.54, *p* = 0.01] and a decrease of RT between B5 and B6 (retention) for DD [*Z*(24) = 2.25, *p* = 0.024].

### Visuospatial Condition

In the Visuospatial condition, Friedman ANOVA revealed a difference in RT between Block 1 and Block 6 for adults [χ^2^(5) = 18.05, *p* = 0.002, *W* = 0.36], for TD [χ^2^(5) = 30, *p* < 0.001, *W* = 0.30] for DD [χ^2^(5) = 27.66, *p* < 0.001, *W* = 0.23], and for DCD + DD [χ^2^(5) = 11.64, *p* = 0.03, *W* = 0.15].

Wilcoxon post-tests revealed an increase of RT between B4 and B5 (specific learning) for adults [*Z*(10) = 2.70, *p* = 0.006], for TD [*Z*(20) = 2.42, *p* = 0.015], and for DD [*Z*(24) = 3.62, *p* < 0.001].

Wilcoxon post-tests revealed a decrease of RT between B5 and B6 (specific learning) for adults [*Z*(10) = 2.70, *p* = 0.006], for TD [*Z*(20) = 2.98, *p* = 0.002], for and DD [*Z*(24) = 4.00, *p* < 0.001].

Regarding the number of errors, in the letter condition, Friedman ANOVA revealed a difference in the number of errors between Block 1 and Block 6 for DD only [χ^2^(5) = 13.45, *p* = 0.019, *W* = 0.11]. Wilcoxon post-tests revealed an increase of RT between B4 and B5 (specific learning) for DD [*Z*(24) = 2.35, *p* = 0.018] and a decrease of RT between B5 and B6 (retention) for DD [*Z*(24) = 2.27, *p* = 0.022].

### Multiple Regression Results

Table 3 shows the results of the multiple regression analysis. The first regression model between the reaction time in the Letter condition and the seven neuropsychological variables identified the attentional and the visuospatial forward working memory score as a significant predictors [*F*(8,61) = 5.19, *p* < 0.001; see Table 3]. The second regression model between the reaction time in the Visuospatial + Letter condition and the seven neuropsychological variables identified the scores of attentional and tactile recognition of fingers as significant predictors [*F*(8,61) = 7.23, *p* < 0.001; see Table 3]. The third regression model between the reaction time in the Visuospatial condition and the seven neuropsychological variables identified the attentional score as a significant predictor [*F*(8,61) = 7.27, *p* < 0.001; see Table 3 and Supplementary Figures].

**TABLE 3:** Summary of linear multiple regression analysis for the neuropsychological Scores predicting the reaction time of the last block (block 6) in the letter condition (upper table), the Visuospatial + letter condition (middle table) and the Visuospatial condition (lower table).

	Factor	B	SE (B)	β	*t*(61)	*P value*	IC 95% (β)
RT in letter condition	Attentional score (GZ-F)	−2.14	0.55	−0.46	−3.89	<.001	[−0.70 −0.23]
	Visuospatial forward working memory	−7.49	3.47	−0.26	−2.16	.034	[−0.51 −0.02]
	
	*Statistics of model: N* = *70. Adjusted R^2^* = *0.327, F(8,61)* = *5.19, p* < *0.001*						

RT in visuospatial + letter condition	Attentional score (GZ-F)	−1.24	0.32	−0.43	−3.89	<.001	[−0.65 −0.21]
	Tactile Recognition of Digits	−36.64	12.34	−0.32	−2.96	<.001	[−0.54 −0.10]
	
	*Statistics of model: N* = *70. Adjusted R^2^* = *0.419, F(8,61)* = *7.23, p* < *0.001*						

RT in visuospatial condition	Attentional score (GZ-F)	−1.78	0.38	−0.52	−4.64	<.001	[−0.74 −0.29]
	
	*Statistics of model: N* = *70. Adjusted R^2^* = *0.421, F(8,61)* = *7.27, p* < *0.001*						

## Discussion

The first objective of this study was to test whether children with DCD and/or DD presented a procedural learning deficit compared to TD children and healthy adults. To this aim, we have created a serial reaction time task (SRTT) with different types of stimuli (Visuospatial, Letters or Combined stimuli). The second objective of this study was to explore whether neuropsychological variables may be linked to the final performance of the sequence. We tested the correlation between neuropsychological scores and the retention of the sequence regardless the group and the condition.

Firstly, our main result is that healthy adults learned the perceptual-motor sequence in the three conditions, which is not the case for children with or without neurodevelopmental disorders. Hence, contrary to healthy adults whose learning and retention performance do not seem to be influenced by the nature of the stimuli, children with or without neurodevelopmental disorders can improve their performance as a function of the specific stimuli. Secondly, the retention of the sequence is linked with the attentional level.

• **Adults learnt in all conditions but not TD children**

As expected, adults presented specific learning and retention of the sequence in all conditions. A surprising result is that TD children present difficulties to learn the sequence in the VS + L condition. It is interesting to note that RT during learning with VS + L is linked to tactile recognition of fingers: as illustrated in the Supplementary Material, the more the children recognized fingers from tactile stimuli, the faster the RT was in the VS + L condition. This result could suggest that the VS + L condition would require to associate each finger to both a position and a letter. The absence of specific learning of TD children in the VS + L condition is intriguing given that children with neurodevelopmental disorders are able to learn in this condition. It can be interpreted in light of previous results of Lejeune et al. (2015) who found that TD children of 10 years old are more affected by dual information processing than younger children (7 years old). Lejeune et al. (2015) interpreted the larger sensitivity to an interference task as the intervention of high-level explicit processes. It is possible that the presentation of two information (VS + L) could have created an interference task requiring the intervention of effortful explicit processes in our group of TD children (mean age: 10 years old), which is not the case in the groups of children with neurodevelopmental disorders who may use more implicit processes, like younger TD children in the experiment of Lejeune et al. (2015). Our experimental protocol did not allow to test explicit knowledge given that the 3 conditions were learned randomly and implicitly, but further studies could help to test this hypothesis. In all cases, our results give some clues to understand the apparent contradictory results about the development of procedural learning. Indeed, some studies argue in favor of an invariance developmental hypothesis (Thomas et al., 2004) whereas others find an increase in procedural learning with age (Zwart et al., 2019). Our study highlight that the developmental trajectory of procedural learning seems to depend on the type of stimuli available for learning (in our study, with or without letters and visuospatial cues).

• **All children with neurodevelopmental disorders failed to learn with Letters only but were able to learn with the combined Visuospatial and Letters stimuli**

All the three groups of children with neurodevelopmental disorders present either difficulties or a deficit to learn. More precisely, DD and DCD + DD present difficulties to learn according to conditions (no significant changes in RT in some phases of learning in L for DD and L and VS for DCD + DD) while DCD present a deficit to learn in some conditions (no significant changes in RT in any phase of learning in L and VS conditions) (see Table 2).

According to the initial hypothesis of Nicolson and Fawcett (2007, 2011), we predicted that procedural learning in the letter condition would be impaired in DD children, with or without DCD comorbidity. Our results are in discordance with this hypothesis given that all children with neurodevelopmental disorders failed to learn with the letter condition. It is possible that letter condition only need a supplementary effort to associated each letter to a finger, which could be difficult for children with neurodevelopmental disorders. This hypothesis is in line with two others results. Firstly, the errors rate (Supplementary Figure A) show that all the groups with neurodevelopmental disorder made more errors across blocks. Given this, it seems that the disordered groups difficulty on the letters version likely reflects a basic problem with performing the task, rather than with procedural memory. Indeed, this might suggest an explicit or associative memory deficit in this group. Secondly, our results highlight that the score of visuospatial forward working memory was a predictor of retention in the Letter condition. The children who present the lower score of visuospatial forward working memory are those who present larger RT during retention in the Letter condition. Even if this hypothesis should be tested specifically, it is possible that retention of the sequence of letters require to translate letters into visuospatial positions of fingers, hence explaining the link with visuospatial forward working memory.

A more positive result is that all groups with neurodevelopmental disorders are able to learn the sequence when the stimuli are a combination of visuospatial and letter cues (VS + L). As postulated above, it is possible that those children use implicit processes (in accordance with Lejeune et al., 2015). Such implicit process may refer to the dual coding theory (Paivio, 1990) that involves the integration of two distinct subsystems: a verbal system specialized for dealing directly with language and a non-verbal system specialized for dealing with non-linguistic objects and events. The presence of the dual non-verbal and verbal information, being functionally independent, can have additive effects on memorization (Clark and Paivio, 1991). Thus, the presence of both visuospatial and verbal cues at the same time and location could optimize learning.

• **Both DCD and DCD + DD groups failed to learn in the Visuospatial condition contrary to children with DD**

In line with the procedural learning deficit hypothesis (Nicolson and Fawcett, 2007), we expected both the DCD and the DCD + DD groups would present procedural learning deficits contrary to the DD group. In accordance with this hypothesis, DD children succeeded to learn the perceptual-motor sequence with the visuospatial stimuli. This result is in accordance with previous results showing an intact procedural learning of a perceptual-motor sequence in DD (Deroost et al., 2010; Menghini et al., 2010; Yang et al., 2013; Vakil et al., 2015; Staels and Van den Broeck, 2017; West et al., 2018). It would be interesting to explore cerebral correlates of learning in order to test whether children with DD achieve learning thanks to higher brain activations than TD children. In fact, Yang and Bi (2011) have shown a learning-related overactivation in the left cerebellum in DD compared to controls.

As regard to the DCD group, our results are in accordance with this hypothesis and in line with previous results of Gheysen et al. (2011) who found a deficit in learning in DCD using a bimanual SRTT. They are also in line with those of Cignetti et al. (2020) who suggest that DCD but not DD present an impairment of cortico-subcortical functional circuits. Given that, compared to TD children, DCD children present abnormalities in cortico-cerebellar connections targeting sensorimotor regions (Cignetti et al., 2020) and less cerebellar activations (Zwicker et al., 2011; Debrabant et al., 2013), it is not surprising that DCD children failed to learn the perceptual-motor sequence in the visuospatial condition. Hence, learning of a bimanual sequence seems to be definitely affected in DCD (see also Blais et al., 2018), which does not seem to be the case for unimanual sequences (Wilson et al., 2003; Lejeune et al., 2013). Lejeune et al. (2013, page 1979) proposed that “*the problem in DCD during the SRT task could be a motor planning deficit rather than an inability to detect and learn the statistical regularities in sequential material.*” However, we cannot say that DCD have a fundamental problem with improving their ability to provide a manual response to the stimulus since they do not show a deficit in the VS + L condition.

As regard to the effect of comorbidity DCD + DD, our results suggest that comorbidity has no adverse effect on procedural learning. On contrary, our results of the 3 phases of learning in the L and VS conditions indicate that DCD present a real deficit in learning these conditions while DCD + DD present only difficulties in learning these two conditions (see Table 2). A study by Biotteau et al. (2015) tested explicit procedural learning in the DCD and DD by measuring the performance in dual task before and after the practice of the explicit learning of an unimanual motor sequence. All children were able to automatize the sequence, suggesting that DCD + DD comorbidity does not constitute an aggravating factor. However, the authors found that DCD + DD children had a behavioral learning profile very close to that of the DD group, and clearly different from that of the DCD group which is opposite to our results. The level of the motor control required to succeed the task (unimanual vs. bimanual) and/or the nature of the instructions and feedbacks (implicit or explicit) could explain this apparent discrepancy, suggesting that comorbid group may present deficits similar either to the DCD group or to the DD group.

• **Procedural learning is mainly linked to the level of attention**

Our second aim was to test the link between neuropsychological variables and procedural learning, as assessed by the retention of the sequence. We expected that neuropsychological variables would relate to the final performance of the learning (retention) of the perceptual-motor sequence regardless the group and the condition, with the assumption that working memory and attention would be important cognitive processes for motor learning (Halsband and Lange, 2006). More exploratory, we have tested the possible link with other neuropsychological variables as visuospatial abilities or tactile recognition of digits. Our results revealed a significant correlation between attentional score and retention of the sequence. The correlation with forward working memory and tactile recognition digits were condition dependent.

Surprisingly, our results did not reveal a link between all the final performance of the sequence learning and the score of the visuospatial working memory. An explanation could be the implicit nature of the SRTT. Indeed, Jongbloed-Pereboom et al. (2019) found that the score of visuospatial working memory positively influenced the retention of the perceptual-motor sequence in an explicit condition, but not in an implicit condition. More precisely, participants with better visuospatial working memory performed faster and more accurate the retention of the perceptual motor learning task. To get an idea of the implicit or explicit nature of the task, some studies used an explicit generation task of the sequence, at the end of learning. Participants were tested for explicit recall of the sequence by asking them if they had noticed a sequence. If so, they were asked to reproduce the sequence (Gheysen et al., 2011; Lejeune et al., 2013). The results of Lejeune et al. (2013) showed that the degree of knowledge of the sequence was low but significant, and that it was similar between the DCD and control groups. However, no relationship was found between explicit knowledge of the sequence and the rate of procedural learning. Thus, although the children (with or without DCD) had some knowledge of the repeated sequence at the end of the learning phase, they did not use this knowledge to increase their performance (Lejeune et al., 2013). Our experimental protocol did not allow to test explicit knowledge given that the 3 conditions were learned randomly and implicitly. However, we waited the very end of the experiment (after practicing the 3 sequences) to ask children whether they had perceived a repetition in the tapping. Their answers were not consistent and it was difficult to answer for the child. Nevertheless, it would be interesting in the future to know if the sequence is explicitly memorized because this result could be related to the link with the visuospatial working memory.

## Conclusion

In conclusion, this study helps to identify the differential deficits in implicit procedural learning in neurodevelopmental disorders and to highlight the condition that could optimize procedural learning. Despite the small sample size in part due to the absence of comorbidities, our results need further studies on procedural learning in DCD and or DD. Globally, children did not present a learning deficit in absolute terms: the deficit was relative to nature of stimuli available to learn the sequence. Our results bring clues to identify environmental cues that could help children with neurodevelopmental disorders and to find adequate solutions to procedural perceptual-motor learning deficit. More specifically, DCD children and DCD + DD children benefit from both letter and visuospatial information whereas DD children benefit more from visuospatial information alone or both letter and visuospatial information. Also, we found that the attentional score is correlated to procedural learning of the motor sequences. Further studies are needed to better understand the procedural perceptual-motor learning of motor sequence by studying cerebral correlates and more precisely the implication of the cortico-striatal network, known as a major network implied in this process.

## Data Availability Statement

The raw data supporting the conclusions of this article will be made available by the authors, without undue reservation.

## Ethics Statement

The studies involving human participants were reviewed and approved by Inserm, 2014-AO1239-38. Written informed consent to participate in this study was provided by the participants’ legal guardian/next of kin.

## Author Contributions

YC, J-MA, MJ, and JT conceived the project and obtained the financial support for this experimentation. MB and JT conceived and planned the experiment, analyzed the results, and wrote the manuscript. MB and SM carried out the experiment. All authors provided critical feedbacks on the manuscript.

## Conflict of Interest

The authors declare that the research was conducted in the absence of any commercial or financial relationships that could be construed as a potential conflict of interest.

## Publisher’s Note

All claims expressed in this article are solely those of the authors and do not necessarily represent those of their affiliated organizations, or those of the publisher, the editors and the reviewers. Any product that may be evaluated in this article, or claim that may be made by its manufacturer, is not guaranteed or endorsed by the publisher.

## Footnotes

1
www.neurobs.com
