# Supplementary material for: Specific Cues Can Improve Procedural Learning and Retention in Developmental Coordination Disorder and/or Developmental Dyslexia
Source: Front Hum Neurosci. 2021 Dec 15;15:744562. doi: 10.3389/fnhum.2021.744562 (PMC8714931; doi:10.3389/fnhum.2021.744562)
Supplement: Supplementary file 1 [file Data_Sheet_1.pdf]

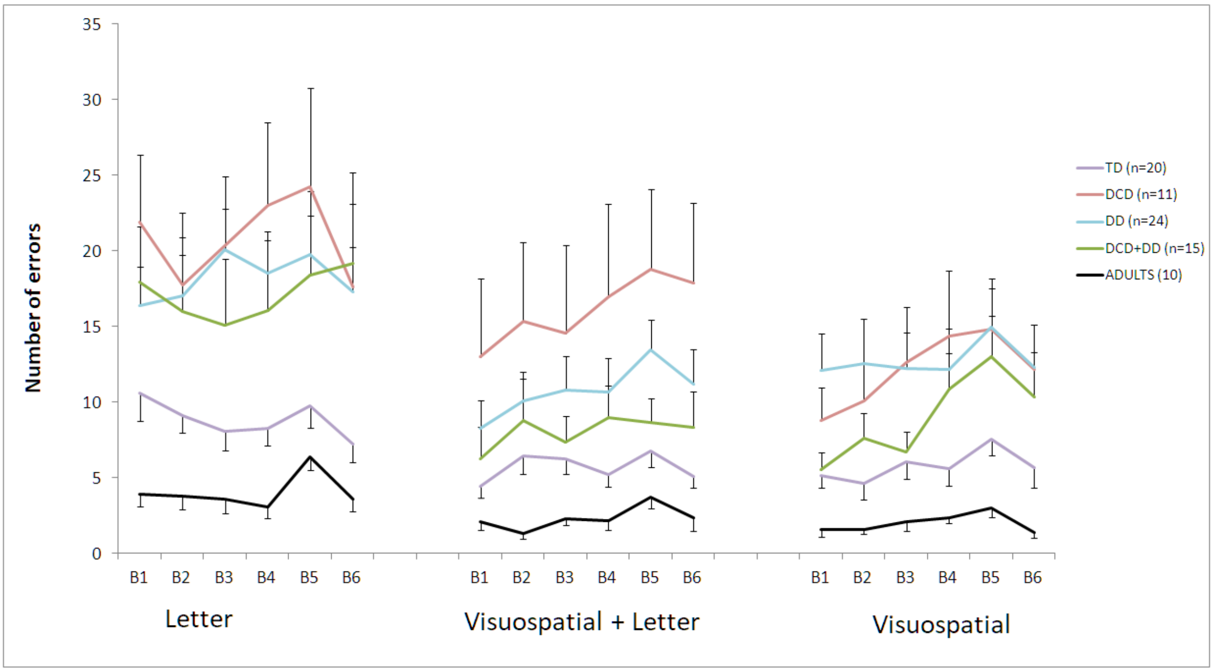

Figure A : Mean number of errors of adults (black), TD group (purple), DCD (red), DD (blue) and DCD+DD (green) groups for Block 1 to Block 6 on Letter condition (left) Visuospatial + Letter condition (middle) and Visuospatial condition (right). Vertical bars represent inter-individual variability (standard errors).

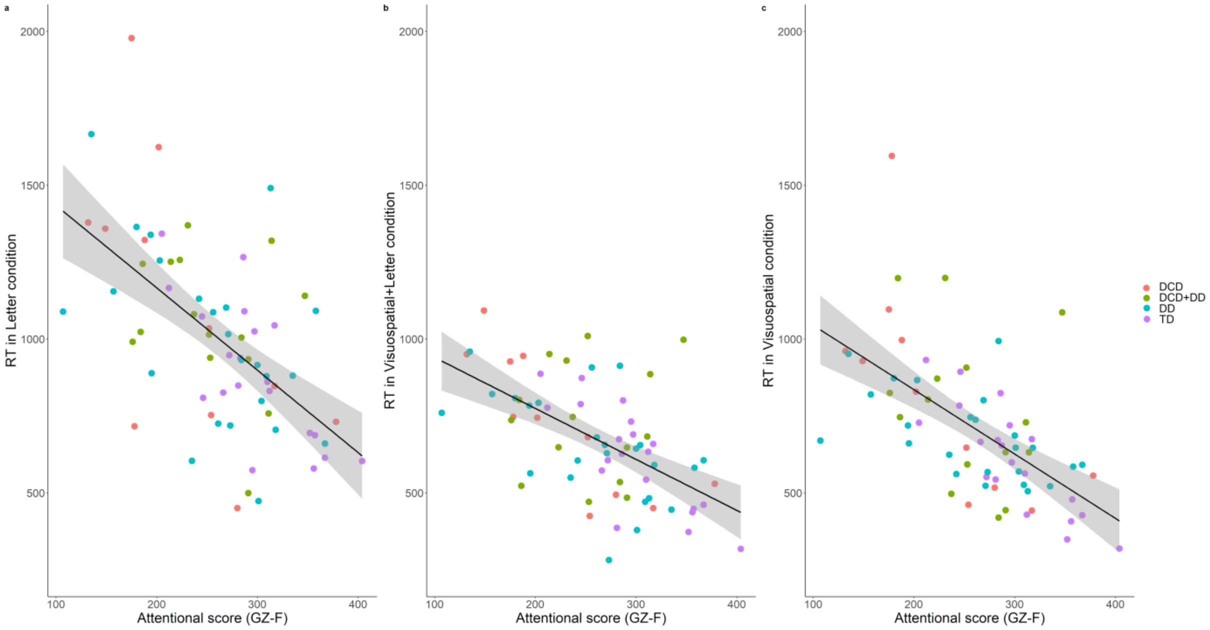

Figure B. Illustration of the link between the reaction time and the Attentional score (GZ-F) (a) in Letter condition (b) in Visuospatial + Letter condition and (c) in Visuospatial condition.

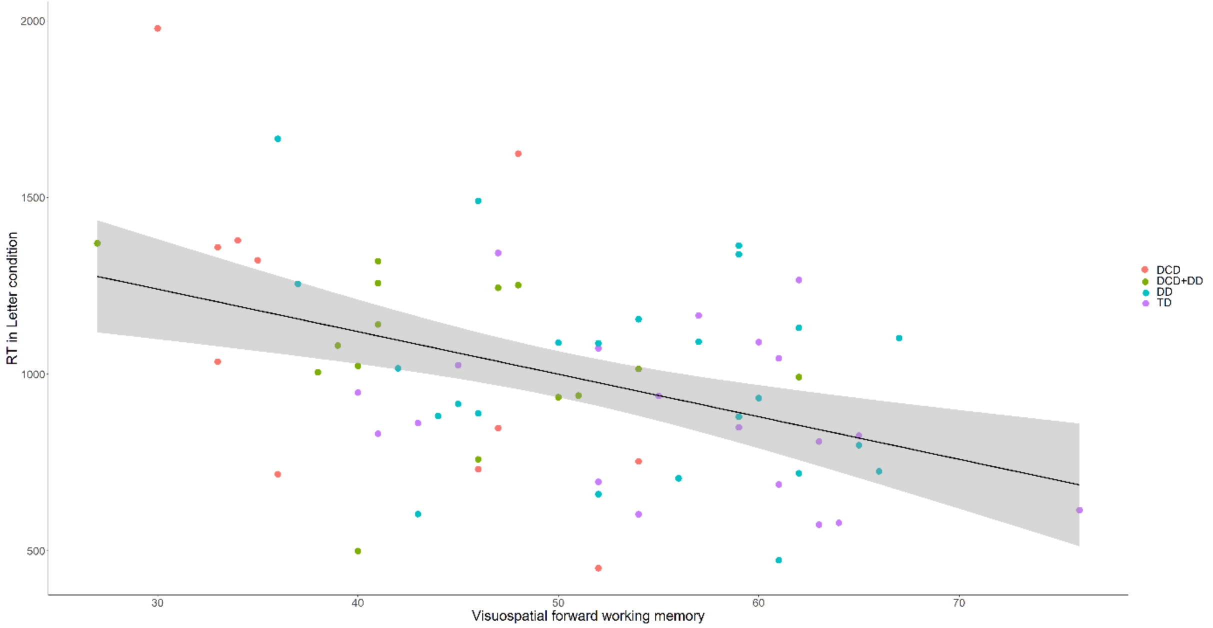

Figure C : Illustration of the link between the reaction time and the Visuospatial forward working memory in the Letter condition.

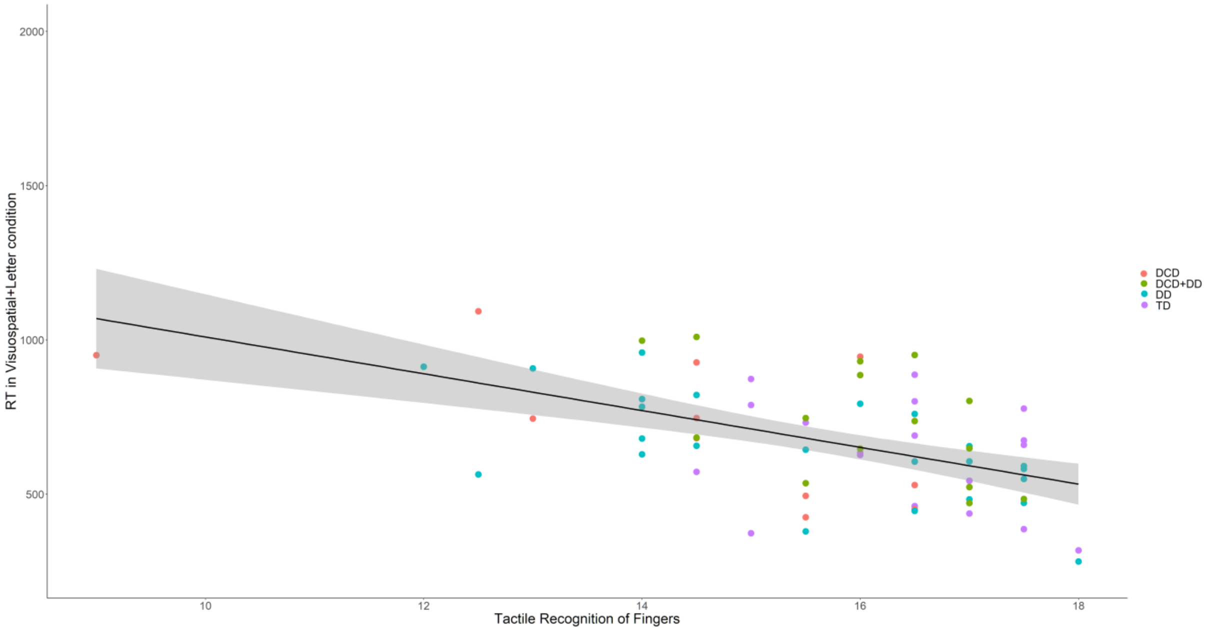

Figure D : Illustration of the link between the reaction time and the recognition of fingers in the Visuospatial + Letter condition.
